# Taylor’s rule, political cycle, and Latin America—An analysis of time series in search of responsibility for monetary stabilization

**DOI:** 10.1371/journal.pone.0259314

**Published:** 2021-12-02

**Authors:** Nadja Simone Menezes Nery de Oliveira, Paulo Reis Mourao

**Affiliations:** 1 Departamento de Economia & GERA, Universidad del Valle (UNIVALLE), Cali, Colombia; 2 Departamento de Economia & NIPE, Universidade do Minho, Braga, Portugal; Istanbul Medeniyet University: Istanbul Medeniyet Universitesi, TURKEY

## Abstract

The decades before 1990 were dramatic for Latin American economies. However, from 1990 onwards, a set of policies followed by the various states in the region acheived economic stabilization with real income recovery. The attribution of this success has been disputed by politicians, economists and officials from international economic support institutions. This work will analyze the responsibility for this success in 4 economies in the region (Brazil, Colombia, Mexico and Peru). Through the combined analysis of ARDL, Markov states and structural breaks, we highlight different sources of responsibility in different periods. Additionally, detailing the states of each regime, we verify the duration of the regimes related to inflation rates and to interest rates in the region. We identify specific governments as associated with moments of economic stabilization in the region, so the hypothesis of the political cycle cannot be rejected for the set of results achieved. As policy implication, we claim that Taylor rules are endogenous to Political Budget Cycles and so stabilization plans are restricted to political tenures.

## Introduction

Several studies on the economic and financial dimensions of Latin American economies have been conducted to assess the Taylor rule because with hyperinflationary episodes in the recent past, these economies have seen their respective national authorities resort to instrumentalizing the interest rate as response to hyperinflation. In this sense, Taylor’s rule (1993) [1] has been the most used functional form to analyze how the monetary authority adjusts the policy instrument: the interest rate. Regarding monetary policy, studies have focused on analyzing how policymakers react to changes in relevant economic variables, as well as on estimating their preferences on certain objective variables, such as inflation and output (Gozgor, 2012; Oliveira *et al*., 2013) [2,3]. However, most of these studies have not properly analyzed the political and electoral cycles in each country with the necessary attention. Some exceptions are Sachs (1989), Stein and Streb (2011) and ECLAC (2003) [4–6]. In any case, the premise that different governments elicit different reactions has not been properly tested for Latin American economies. The consideration of different governments, with their own compositions, with their own dynamics of economic and financial policies, and with specific international policy frameworks, matters in the analysis of the problem of conducting national monetary policies.

Given the incidence of the problem, studies such as those by Rodriguez (1994), Rojas-Suarez and Sotelo (2007) and Luo (2013) [7–9], to name a few on a longer wider list, have focused on the evolution of interest rates or inflation in Latin American economies in considerable detail. Rodriguez (1994) and Rojas-Suarez and Sotelo (2007) [7,8] studied interest rates, while Haberler (1974) and Payer (1974) [10,11] analyzed inflation rates in several episodes and periods dating back to 1960.

This work will thus test the economic significance of the political cycle for the Taylor rule in four Latin American economies: Brazil, Colombia, Mexico, and Peru (which represent 70% of the total of the gross domestic product of the Latin American economies). Observing the evolution of the interest rates as a function of the profile of the general price level and the GDP gap, we will verify whether the evolution of the inflationary processes in the region due to the instrumentalization of the interest rate was the responsibility of certain governments or, on the contrary, if it was due to a political process that involved several legislatures. Thus, we understand that the main contribution of our work to the existing literature consists in the fact that we consider the premise that different governments may provoke different reactions in the conduct of monetary policy in developing economies, such as the case of Latin American economies.

The structure of the paper is as follows. Section 2 reviews the literature while Section 3 empirically analyzes the existence of Taylor’s rule on Latin America, recurring to the combined discussion of Autoregressive Distributed Lags, Structural Breaks and Cointegration Analysis. Section 4 concludes.

## Literature review—Latin American economies meandering between Taylor’s rule and the right governments at the right time

This section presents the main financial and structural reforms that took place in the 1990s in Latin America and in the four countries that are the object of our study. To this end, the main reforms and their impacts on these economies are revisited from studies carried out for this period.

### Latin American reforms and governments since 1980

Structural reforms took place in Latin American countries in the 1990s, a period characterized by profound economic transformations in several countries in this region. During this period, economic policies were implemented and were justified as an instrument to combat the effects of the economic crisis that appeared in the region in the 1980s. Essentially, these changes were aimed at reducing state intervention in the economy and strengthening the market as a provider of goods and services (Bresser-Pereira, 1996; Przeworski, 1996; Almeida, 2005) [12–14].

Specific economic policies, which consisted of institutional reforms, were implemented in the region between the late 1980s and the 1990s. The political framework was developed in the mid-1980s by the Institute for International Economics in Washington with the goal to achieve a new way of growth for Latin America based on ten topics: Increase in savings through strong fiscal discipline; Reorienting public spending towards well-designed social programs; Reform of the tax system to expand the tax base; Consolidation of Central Bank supervision; Conservation of (flexible) exchange rate; Liberalization of intraregional trade; Generation of a competitive market economy through privatization and liberalization of goods and services markets, with special emphasis on the deregulation of the labor market; Safeguarding property rights for society as a whole; Creation of an autonomous central bank, independent and incorruptible judiciary, and entities that promote productivity; Increase in public spending directed to primary and secondary education.

Following the prescription of dominant policies, in the course of the 1990s, most countries made a commitment to opening their economies abroad. In addition, significant advances were made with regard to privatization, albeit with wide variations from one country to another. In general, the stabilization and privatization processes were carried out quickly, while financial, labor market and social assistance reforms were carried out gradually. Trade liberalization and tax reform were intermediate cases, in which neither a gradual approach nor a shock treatment was the rule (Edwards, 1995) [15].

These efforts have had positive results, including a general decline in inflation rates, a relative recovery in the balance of payments, and partial monetary policy credibility. However, in most Latin American countries, the adoption of these structural reforms was preceded by the deterioration of economic conditions, with declining investment rates, low or falling economic growth rates, large fiscal deficits, accelerated inflation and a sharp contraction in financial intermediation (Carneiro; Rocha, 2000) [16].

Below, we present a comparative summary of the financial and structural reforms of the four Latin American economies analyzed in this study: Brazil, Colombia, Mexico, and Peru (Table 1).

**Table 1:** Main structural reforms in Brazil, Colombia, Mexico, and Peru.

**Country**	Structural reforms	Impacts	References
**Brazil**	The economy’s trade opening process (1992)	GDP increased by 21.6%, or 4% per year. Initially exchange rate appreciation. Involving a trade balance deficit (1995–1998).	[17–21]
	Privatization process for state-owned companies (1990’s)	Important sectors of the economy, such as transport, telecommunications, and electricity, were partially or completely transferred to the private sector.	
	Real Plan (1994)	Elimination of the biggest problem in the Brazilian economy until 1994: the phenomenon of “super inflation”. Stabilization of the Brazilian economy.	
	Change in the exchange rate regime, with the introduction of free flexibility (1999)	Recovery of monetary policy credibility.	
	Inflation targeting regime (1999)	Control of the inflation rate.	
**Colombia**	The economy’s trade opening process and the beginning of the autonomy of Banco da República (1990’s)	The central bank started operating with inflation targets. Reduction in inflation for rates below 10%. Privatization of state-owned companies. Reduction in public spending. Elimination or reduction of social benefits.	[22–26].
	Exchange rate fluctuation (1999)	Maintaining a stable exchange rate. Reduction of inflation to single-digit levels. Relative stability of the exchange rate.	
	Inflation targeting regime (2000)	Multiannual announcement of inflation targets. Global assessment of the macroeconomic environment, especially the situation of the real and financial sector. Strengthening of the Central Bank’s operational instruments through the adoption of monetary signs via interest rates. Adoption of the "put" option system to accumulate or "call" to de-accumulate international reserves.	
**México**	Financial liberalization (19980)	Liberalization of interest rates. Replacement of the legal reserve with a liquidity index. Bank privatization. Central Bank autonomy. Control of inflation, interest rates and exchange rates and redefinition of the State’s participation in the economy	[27,28].
	The economy’s trade opening process, trade reform, (1988–1994)	Privatization of state-owned companies. Higher levels of efficiency and modernization of the Mexican economy.	
	Welfare, Stability and Growth Pact (PBEC). Unit Agreement to Overcome Economic Emergency (AUSEE). Program for Strengthening the Economic Emergency Agreement (PARAUSEE) (1988–1994)	Stabilize the economy from the effects of the 1994–1995 and 2001–2003 and 2008 crises. Deepening trade liberalization.	
**Peru**	The economy’s trade opening process (1991)	Increased competitiveness of the tradable sector of the economy. Limiting the increase in domestic prices of tradable goods. Reduction of inflation. Increased tax revenue.	[29,30].
	Opening of the capital market (1991)	Liberalization of the country’s capital flows abroad. Free maintenance of foreign currency accounts. End of the need to report foreign exchange movements to the Central Bank of Peru (BCRP).	
	Financial reform (1990’s)	Elimination of financial repression. Development of the capital market and reduction of transaction costs in financing operations.	
	Labour reform (1990’s)	Reduction in hiring and firing costs. Flexibility of wages, hours, and types of employment contracts. Expansion of contracting modalities for a fixed period.	

**Source**: Prepared by the authors based on related literature.

### Economic and social development results

In the 1990s, total production in the region increased by 3.6% during the first part of the decade, domestic demand increased by 4.4%, investment increased by more than 8%, and exports tended to increase permanently, despite imports presenting lower growth rates (Reyes, 2000) [31].

**Fig 1:**
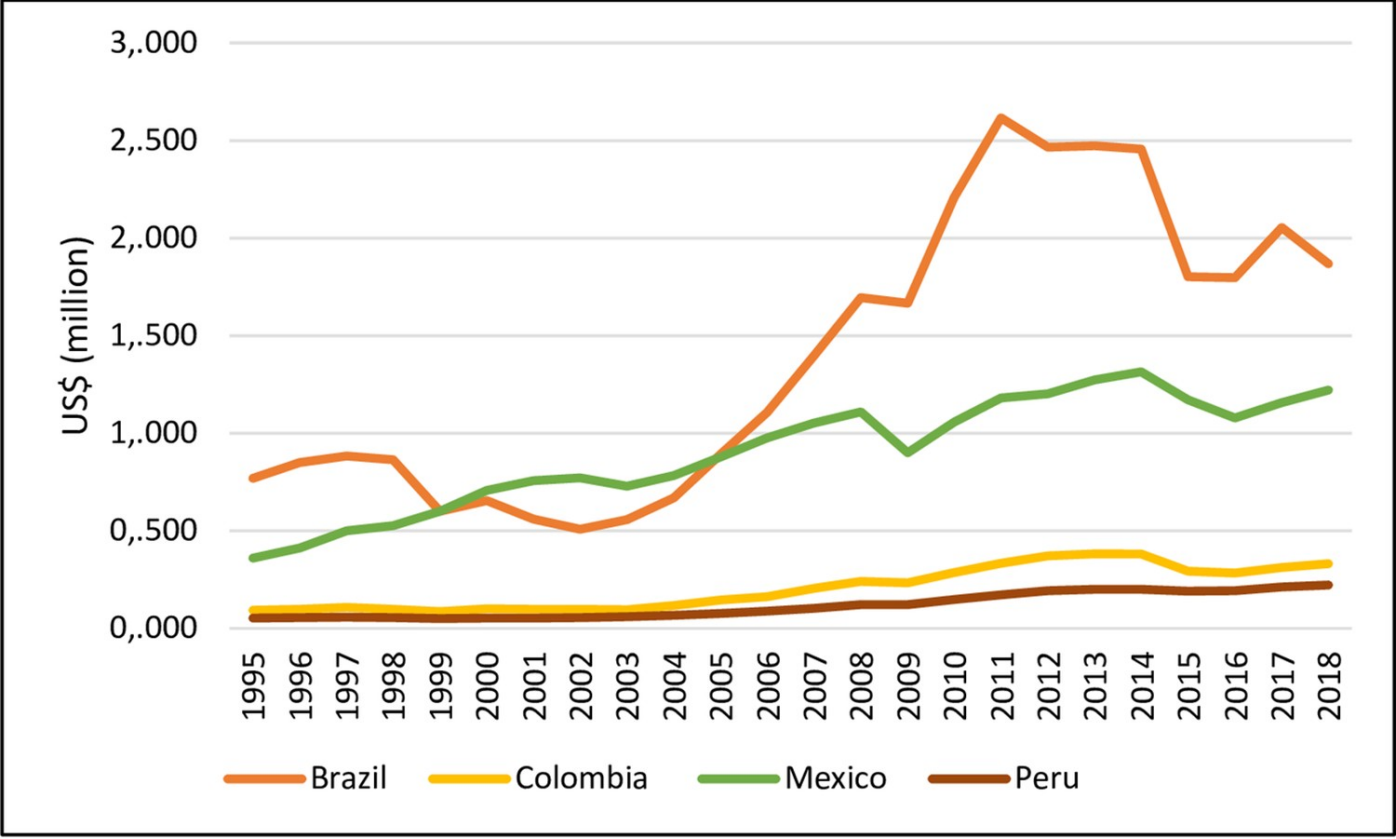
Gross Domestic Product at current prices (1995–2018). Source: World Bank (2020).

Fig 1 shows the performance of GDP for Brazil, Colombia, Mexico, and Peru in the period from 1995 to 2018. As noted, Brazil and Mexico have the highest GDP. In Brazil, there is also a reduction in GDP growth, especially since 2015, breaking with the growth trajectory observed from 2003 to 2013. Since 2016, Colombia, Mexico and Peru have shown a relative increase in their GDPs.

The year 2003 is recognized as the first of the last major booms in Latin American economies. This boom was, in part, a consequence of internal factors. The first factor was the natural recovery of production. In addition, economies had already undergone a process of "partial modernization". On the other hand, democratic advances created a better political and social climate (Gasparini et al., 2011) [32].

There has been a high level of concentration of economic power in the region and a social system based mainly on the concentration of incomes. Inequality has been a historical and structural feature of Latin American societies that has remained and been reproduced even in periods of economic growth and prosperity (ECLAC, 2016) [33].

**Fig 2:**
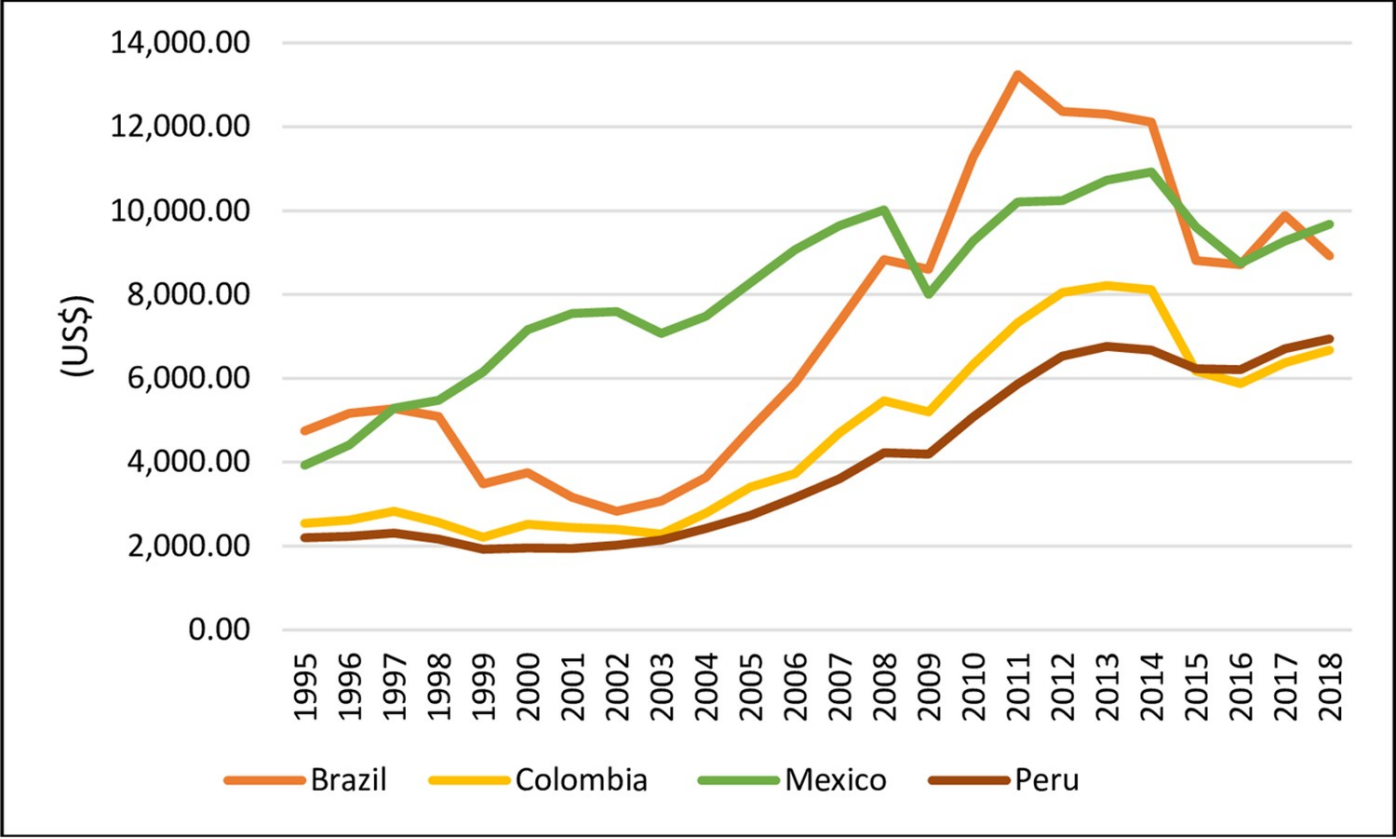
Gross Domestic Product *per capita* at current prices (1995–2018). Source: World Bank (2020).

Fig 2 shows the performance of GDP per capita for Brazil, Colombia, Mexico and Peru in the studied period from 1995 to 2018. As noted, Brazil and Mexico have the highest GDP per capita. These countries show a trajectory of relative growth in their GDP per capita (starting in 1998 in Mexico, in 2003 in Brazil and Peru and in 2004 in Colombia). This trajectory was clearly interrupted in 2009 as a consequence of the financial crisis of 2008.

**Fig 3:**
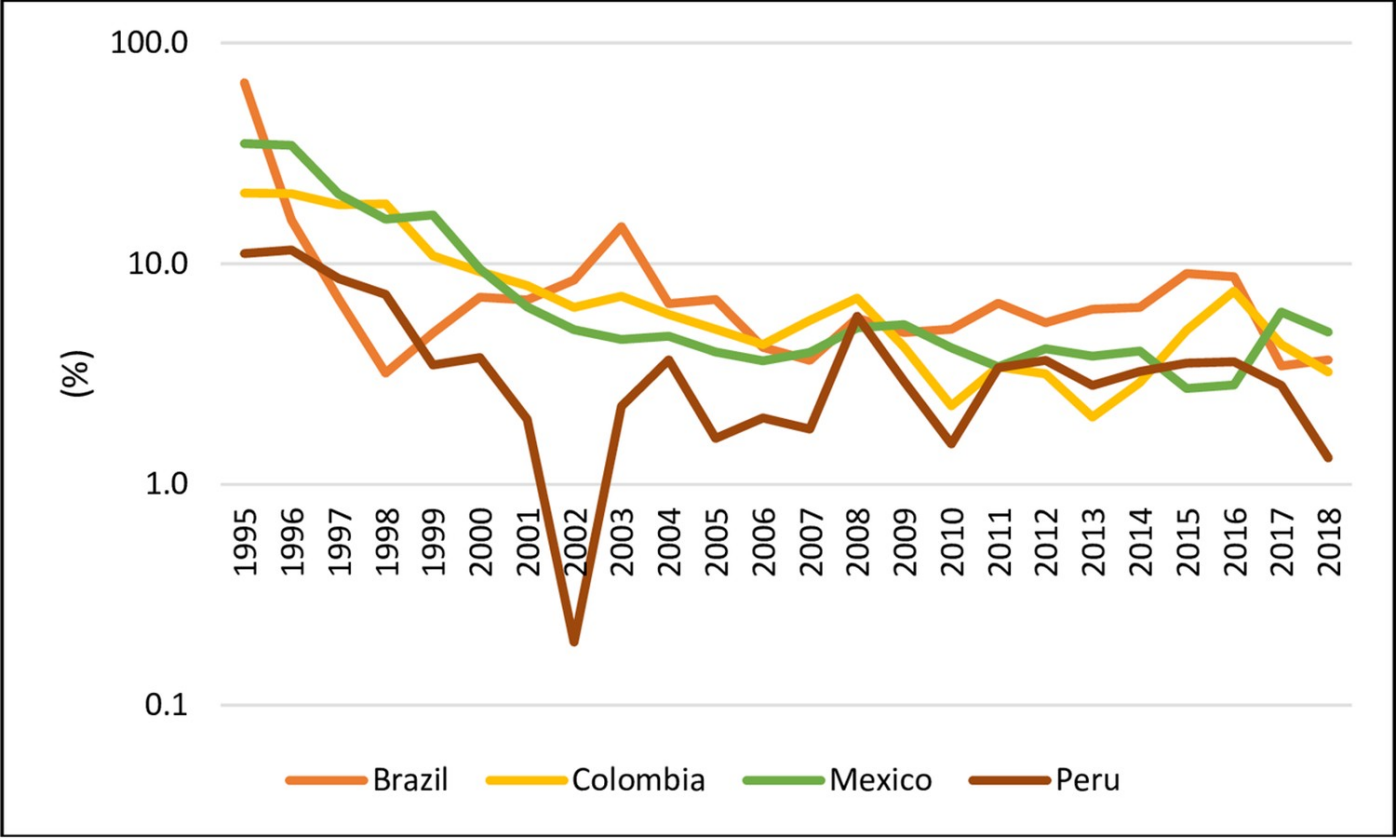
Inflation rates, yearly (1995–2018). Source: World Bank (2020).

Fig 3 shows the inflation rates of Brazil, Colombia, Mexico, and Peru in the period studied from 1995 to 2018. As noted, Brazil and Peru showed the greatest fluctuations. Until the end of the 90’s, these countries showed a trajectory of intense fall in their inflation rates. In the case of Colombia and Mexico, this downward trajectory was observed until the year 2006. At the beginning of the 2000s, Brazil (14.7% in 2003) and Peru (0.2% in 2002) presented their peaks of high and lower its inflation rates. However, we want to analyze whether these trajectories have been influenced by national political cycles which additionally conditioned the profile of the respective plans of stabilization. Next section will test these hypotheses.

## Empirical analysis of the political cycle on the Taylor rules in South America

This section will exhibit the empirical analysis of the political cycle on the monetary policies of 4 South-American economies, stabilized through the Taylor-rule mechanism. We will briefly introduce the regional debate considering focused literature and then in a subsequent sub-section we will exhibit the methodological procedures based upon ARDL estimations and upon Markov-Switching models.

### Taylor’s rule—An old debate that is not yet over in Latin America

In his influential article, John B. Taylor (1993) [1] presented a simple linear equation between the behavior of domestic short-term interest rates in the United States, the deviation of inflation from an established inflation targets and the deviation of real output in relation to the potential product for the period from 1987 to 1992. Taylor sought to show that monetary policy should be conducted through transparent and credible rules, since he believed that this was the most effective way to achieve the best joint economic performance results. According to Taylor (1993) [1], the performance of interest rates in the USA could be represented by a simple linear relationship with the inflation rate, an equilibrium interest rate, and a weighted sum between two deviations: the difference between the interest rate inflation (measured by the GDP deflator) and the inflation targets and the percentage of deviation in GDP from its potential. This linear relationship can be expressed as

$$i_{t}=\pi_{t}+r^{*}+0,5(\pi_{t}-\pi^{*})+0,5(y_{t})$$ (Eq 1)

where i_t_ is the interest rate, r_t_ * is the equilibrium real interest rate, π _t_ is the inflation rate (measured by the GDP deflator), π * is the inflation target, and y_t_ is the output gap (that is, the percentage deviation of the actual product from the potential product).

From the work of Taylor (1993) [1], numerous studies, both theoretical and empirical, have been carried out to detail the suggested relation. Among these works, we highlight the one of Clarida, Galí and Gertler (1998) [34] who estimated reaction functions for central banks in industrialized countries. The authors divided these countries into two groups, the G3 (Germany, Japan, and the USA) and the E3 (Italy, France, and the United Kingdom). This study showed that the central banks of the G3 group have a forward-looking perspective, responding to the expected inflation as opposed to the backward-looking perspective, adopted by Taylor (1993) [1], where the past values of inflation and output were used in the model.

The basic rule estimated for each country is given by:

$$r_{t}=(1-\rho )\alpha +(1-\rho )\beta \pi_{t+n}+(1-\rho )\gamma x_{t}+\rho +r_{t-1}+\varepsilon_{t}$$ (Eq 2)

where r_t_ is the nominal interest rate, π _t + n_ is the inflation rate in the period t + n, x_t_ is the output gap and ε_t_ is the error term.

These authors concluded that the countries which compose the G3 group have implicit inflation targets. Considering the sample data, they explicitly argue that an inflation targeting system is preferable to a fixed exchange rate system, in terms of the joint performance of inflation rates and economic growth.

Aragón and Portugal (2009) [35] check whether the effects of monetary policy actions on output in Brazil were asymmetric. For this, they estimated Markov-switching models allowing for positive and negative shocks to affect the growth rate of output in an asymmetric fashion in expansion and recession states.

**Fig 4:**
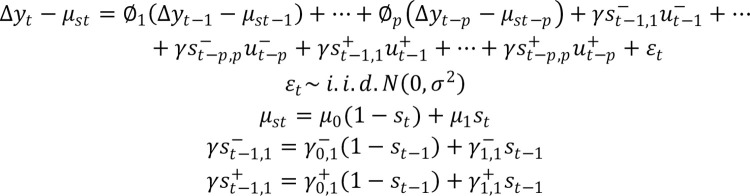
Specification of the Markov-switching model.

The basic specification of this Markov-switching model was given by Fig 4.

where Δ*yt* is the growth rate of the real Brazilian GDP, μ*_st_* is the mean state-dependent growth rate of output, u^-^_t-i_ is a negative monetary shock, γ^-^_St-i,i_ is the state dependent coefficient measuring the response of Δ*yt* to a negative monetary shock, *u_i_* ^+^ is a positive monetary shock and γ^+^_S t-1,i_ is the state-dependent coefficient measuring the response of Δ*yt* to a positive monetary shock. The state variable St assumed the value 0 when the economy was in a recession and value 1 when it is in an expansion. Thus, the model parameters in a recession are μ_0_, γ^-^_o,*i*_ and γ^+^_0,i_, whereas, in an expansion, these parameters are given by μ_1_, γ^-^_1,*i*_ and γ^+^_1,*i*_.

The results showed that: i) the real effects of negative monetary shocks are larger than those of positive shocks in an expansion; ii) in a recession, the real effects of positive and negative shocks tended to be the same; iii) there is no evidence of asymmetry between the effects of countercyclical monetary policies; and iv) it is not possible to assert that the effects of a positive (or negative) shock are dependent upon the phase of the business cycle.

Liu, Waggoner and Zha (2011) [36] proposed an efficient methodology for estimating regime switching DSGE models to examine the sources of macroeconomic fluctuations by estimating a variety of richly parameterized DSGE models within a unified framework that incorporates regime switching both in shock variances and in the inflation target. The authors’ counterfactual exercises showed that changes in the inflation target are not the main driving force behind high inflation in the 1970s. The model that best fits the U.S. time-series data is the one with synchronized shifts in shock variances across two regimes, and the fit does not rely on strong nominal rigidities. They evidence that a shock to the capital depreciation rate, which resembles a financial shock, plays a crucial role in accounting for macroeconomic fluctuations.

Gozgor (2012) [2] estimates reaction functions of the Central Bank of the Republic of Turkey (CBRT) based on the Taylor rule and the Hybrid McCallum-Taylor rule. For this, and with the objective of estimating the monetary policy reaction functions during the period in which the CBRT conducted inflation targets using the nominal interest rate as a monetary policy tool in the free-floating exchange rate, the author uses methods generalized moments (GMM) and limited information maximum probability (LIML) methods. The results found show that only the Taylor rule specifications can explain the behavior of the CBRT.

Oliveira et al. (2013) [3] tested the hypothesis of the occurrence of possible points of structural changes, that is, changes in the dynamics of the definition of the Selic rate by the Central Bank of Brazil (BACEN) in the period 2000 to 2011. Let us notice that Selic is Abbreviation for Special Settlement and Custody System. To this, they used the methodology proposed by Bai and Perron (2003) [37], which consists of estimating the occurrence of more than a structural break point at an unknown date. The results obtained show that since the adoption of the inflation targeting regime, the coefficients of the monetary policy rule adopted by BACEN during the 2000–2011 period have not remained constant. The structural changes test indicated that there is evidence of two structural breaks in the Central Bank of Brazil reaction function coefficients in the inflation targeting period, the dates of the structural changes obtained were 2004.2 and 2007.10. The authors also found that the Brazilian monetary authority has reacted positively to deviations in inflation expectations in relation to its target and to the output gap, having been more sensitive to deviations of inflation expectations in relation to the inflation target.

Nevertheless, Taylor’s (1993) [1] article would gain increasing popularity in the subsequent decades. The explanations for this notoriety were discussed in Orphanides (2010) [38] and range from the Taylor rule filling a space left by criticisms of the Phillips Curve (Barbosa, 2018) [39] to the need to stabilize economies under inflationary shocks (Dedes, 2018) [40].

In particular, as Payer (1974) and Haberler (1976) [41,42] debated, since the 1960s, Latin America has been involved in several episodes characterized by rising inflation, which in many cases have been identified as hyperinflationary. The various governments in the region have tested the use of various monetary, fiscal and exchange rate policy instruments (Sargent et al., 2009) [43]. External intervention has also been present in several Latin American cases that have resorted to the intervention of the International Monetary Fund since 1980 (Portella Filho, 1994) [44].

**Fig 5:**
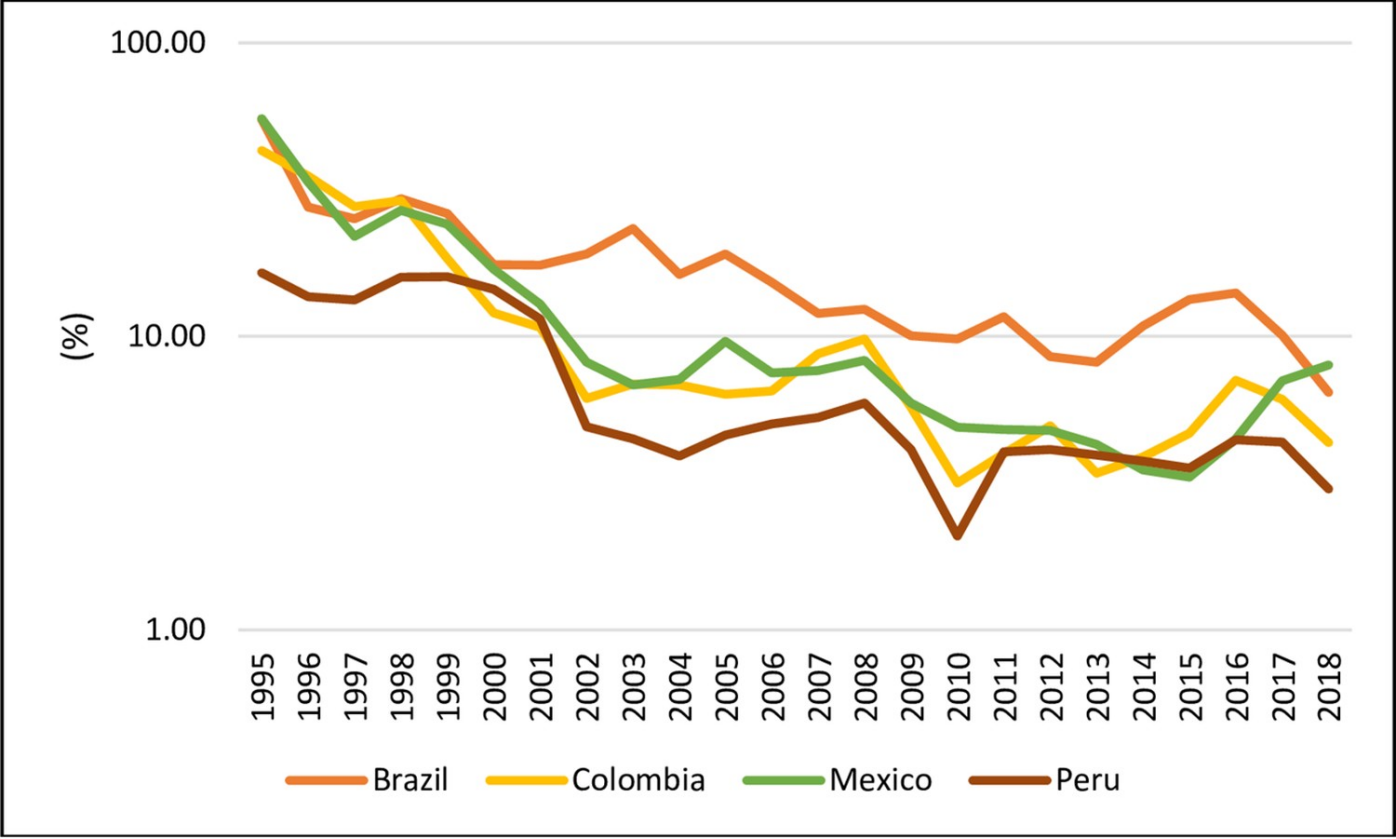
Nominal Interest rates, yearly (1995–2018). **Source**: Banco Central do Brasil (2020); Banco de la Republica Colombiana (2020); Banco de México (2020); Banco Central de Reserva de Perú (2020).

However, as Portella Filho (1994) [44] and Fig 3 show, since the first decade of the 2000s, the values of inflation or of the main interest rates (Fig 5) of each country showed a stabilization movement.

An intense debate soon emerged: Who should receive credit for this stabilization? Authors such as Barajas et al. (2014) and Moura and Carvalho (2009) [45,46] pointed out the role of the application of the Taylor rule by most political and monetary authorities in the region. Other studies, such as Sargent et al. (2009) [43], identified the main role of external intervention and the exogenous evolution of international markets as the main factors responsible for this stabilization. Within the first line of debate—the one focused on the accountability of national political and monetary authorities—another debate emerged. This debate focused on political cycles and different political generations. Basically, authors such as Reinhart (2013) [47] intended to attribute the credit for the stabilization achieved to the administration of a certain party or political generation. The existence of recent works such as Ozdemir (2015) [48] proves that this debate is not over and that it remains active for three fundamental reasons.

The first reason is related to the need to identify which dominant forces still intervene in the stabilization of economies under inflationary shocks, episodes that still appear with recurrence and with socioeconomic significance (Matolcsy et al., 2020) [49]. The second reason is related to a certain political competence that emerges in the work of Stein and Streb (1997) [5] that associates the merit of stabilization policies with a determined party or political generation in power. Finally, the strength of this debate has reemerged due to works such as those by Horvath et al. (2020) and Mourao and Stawska (2020) [50,51] that seek to reconstruct the evidence of the relationship between inflation and interest rates after 2015; this has emerged as a troubled relationship due to the presence of negative interest rates in many investment markets, which requires a successive formulation of both the Phillips curve and the Taylor rule.

#### Looking for a robust Taylor rule in Latin America – ARDL, error corrections models, structural breaks, and Markov-Switching regimes in the Latin-American series

To test the robustness of these findings, we will use several econometric techniques that are useful for identifying structural breaks in time series and for identifying regime changes in time series. We will explore the time dynamics of the observed series by studying ARDL models as well as their long-run version considering error-correction models assessed by proper bond tests (Pesaran et al., 2001) [52]. Additionally, we will explore the existence of simultaneity between structural breaks in the series and government changes in the studied Latin American economies. We will also use the technique for identifying multiple time breaks, and afterward, we will use “Markov switching models”.

We will start this empirical section by employing autoregressive distributed lags (ARDL) for modeling interest rates in the 4 Latin American countries that we are observing in the period 1995q1–2018q4. Considering this approach, we will also use the bounds testing approach to analyze cointegration (ARDL) proposed by Pesaran et al. (2001) [52]. Extensive literature has shown that the ARDL approach offers statistical advantages over other cointegration techniques (Bloch et al, 2015). While other cointegration techniques require all variables to be integrated of the same order, the ARDL test procedure provides valid results whether the variables are I(0) or I(1) or mutually cointegrated. Additionally, it allows for estimating the long- and short-run relationships between the variables while providing efficient and consistent test results in small and large sample sizes (Pesaran et al., 2001; Dritsakis, 2011) [52,53].

The general form for an ARDL (p, q1, q2, …, qk)—where ‘p’ is the extension of lags of the dependent variable included in the estimation and ‘q1…qk’ is the extension of lags for the k independent variables—is the following:

$$A(L)y_{t}=\mu +B_{1}(L)x_{1t}+\ldots +B_{k}(L)x_{kt}+u_{t}$$

We also define A(L) as the lag polynomial and B(L) as the vector polynomial:

$$A(L)=1-\alpha_{1}L-\alpha_{2}L^{2}-\ldots -\alpha_{p}L^{p}$$

$$B(L)=1-\beta_{1}L-\beta_{2}L^{2}-\ldots -\beta_{p}L^{q}$$

For our purpose, we will observe for each country c the following specification of the respective ARDL [54–56]:

$$A(L)i^{c}{}_{t}=\mu +B_{1}(L)\pi_{t}^{c}+B_{2}(L)y_{t}^{c}+u_{t}^{c}$$

In the previous specification, i represents the interest rates recorded in each economy, π the respective inflation rate and y the respective output gap.

If we model the previous equation ARDL (1,1,1) as an error correction model, we will obtain

$$\Delta i_{t}^{c}=\mu +\beta_{1}\Delta \pi_{t}^{c}+\beta_{2}\Delta y_{t}^{c}-\lambda \left[i_{t-1}^{c}-\varphi_{1}^{c}\pi_{t-1}^{c}-\varphi_{2}^{c}y_{t-1}^{c}\right]+u_{t}^{c}$$

$i_{t-1}^{c}-\varphi_{1}^{c}\pi_{t-1}^{c}-\varphi_{2}^{c}y_{t-1}^{c}$ is the error correction term obtained from the cointegration model. The error correction coefficient (λ) represents the speed of adjustment to restore the long-run equilibrium relationship. A negative and significant coefficient for λ implies that any short-term movement between the dependent and the explanatory variables will converge back to the long-run relationship. The estimated coefficients ß1 and ß2 are identified as short-run coefficients related to the expected changes in the first differences of the dependent variable stimulated by the first differences of each independent variable (i.e., by, respectively, $\pi_{t}^{c}-\pi_{t-1}^{c}$ or $y_{t}^{c}-y_{t-1}^{c}$).

Additional tests can be run for assessing the stability and providing overall diagnoses of the relationships found. The traditional Breusch-Godfrey serial correlation, the White test for heteroscedasticity, and the CUSUM test (or its cumulative version) also employed to verify the stability of long-run coefficients together with short-run dynamics are observed for discussing the quality of the estimated equations.

Table 2 shows the descriptive statistics for our quarterly series and the respective sources. Table 3 shows the ADF tests on our series to discuss their level of integration. We have also tested Advance Unit-Root tests (including structural breaks) and the outcomes converged with the exhibited ones in Table 2. Due to the current extension of the paper, these tests will be available under request. Table 4 exhibits our results from the estimations of ARDL models for the relation between national interest rates and inflation and output gap. Let us also notice that for interest rates we are considering short-term interest rates from the official sources.

**Table 2:** Descriptive statistics (quarterly series, 1990q1 – 2018q4).

Variable	Obs	Mean	Std. Dev.	Min	Max
Brazil (Inflation rates)	288	12.60495	48.25334	1.65	86.54
Brazil (Interest rates)	288	17.4624	10.87455	6.4	75.47
Brazil (Output Gap)	288	0.73201	-115.017	3.2862	27.5137
Colombia (Inflation rates)	288	6.593611	0.656141	-0.32	34.01
Colombia (Interest rates)	288	6.989417	3.696432	1.32	76.65
Colombia (Output gap)	288	7.542639	5.962444	0.63	21.88
Mexico (Inflation rate)	288	20.67944	0.91064	-0.74	37.97
Mexico (Interest rates)	286	12.11129	12.2088	3.287321	89.4825
Mexico (Output Gatp)	288	8.816042	9.708349	2.13	51.97
Peru (Inflation rate)	288	3.303439	-0.37665	0.53798	11.53377
Peru (Interest rates)	288	7.206486	4.987788	1.356305	26.58971
Peru (Output gap)	288	3.914479	3.002274	-1.11436	13.72404

Source: World Bank (2020).

**Table 3:** Augmented Dickey-Fuller (ADF) Statistics of the series studied in this work.

*y_t_*	Δ*^d^y_t_*	ADF		
		No interception; No trend	With interception; No trend	With interception; With trend
Brasil (Inflation rates)	d=0	-2.294***	-3.243**	-4.212***
	d=1	-20.045***	-20.071***	-20.090***
Brasil (Interest rates)	d=0	-81.574***	-103.649***	-101.151***
	d=1	-38.788***	-38.362***	-37.247***
Brasil (Output Gap)	d=0	-1.313	-1.314	-1.309
	d=1	-1.734*	-1.735	-1.729
Colombia (Inflation rates)	d=0	-7.849***	-10.457***	-9.019***
	d=1	-6.510***	-6.548***	-6.574***
Colombia (Interest rates)	d=0	-4.503***	-6.023***	-7.034***
	d=1	-16.549***	-16.530***	-16.519***
Colombia (Output gap)	d=0	-0.741	-0.728	-0.712
	d=1	-1.554	-1.554	-1.547
Mexico (Inflation rate)	d=0	-7.128***	-8.286***	-8.633***
	d=1	-11.898***	-11.970***	-12.196***
Mexico (interest rates)	d=0	-4.498***	-5.395***	-6.010***
	d=1	-18.169***	-18.149***	-18.156***
Mexico (Output Gap)	d=0	-0.741	-0.728	-0.712
	d=1	-1.554	-1.554	-1.547
Peru (Inflation rate)	d=0	-7.420***	-10.140***	-10.785***
	d=1	-23.574***	-23.534***	-23.494***
Peru (Interest rates)	d=0	-1.678**	-1.824	-2.529
	d=1	-13.386***	-13.386***	-13.386***
Peru (Output gap)	d=0	-0.479	-0.481	-0.479
	d=1	-1.403	-1.394	-1.371

Significance level: 1%, ***; 5%, **; 10%, *.

**Table 4:** ARDL results for the relation between national interest rates and inflation and output gap.

	Brazil ARDL (2,1,1)	Colombia ARDL (2,1,1)	Mexico ARDL (2,1,2)	Peru ARDL (1,1,1)
Adjustment term/Error Correction term	-0.054*** (0.020)	-0.034*** (0.009)	-0.161*** (0.031)	-0.021** (0.011)
Long-Run				
Inflation	-2.433* (1.382)	0.882*** (0.207)	0.886*** (0.081)	-7.076* (3.856)
Output gap	-0.008 (0.024)	0.022 (0.023)	0.007 (0.011)	0.398 (0.460)
Short-run				
Inf. rates	0.114** (0.046)	0.509*** (0.046)	0.301*** (0.058)	0.289*** (0.055)
Output gap	0.143 (0.323)	0.229 (0.432)	0.288 (0.461)	0.299 (0.477)
Constant	1.513*** (0.294)	0.058 (0.037)	0.561*** (0.164)	0.126 (0.091)
F(test), sign. level acc PSS(2001)	25.414***	5.006**	10.339***	4.498**
T(test), sign. level acc PSS(2001)	-2.669***	-3.651**	-5.237***	-3.474**
Breusch-Godfrey LM test for Autocorrelation (H0: No autocorrelation); p-values	Lag1: 0.637 Lag2: 0.723 Lag3: 0.776 Lag4: 0.812	Lag1: 0.536 Lag2: 0.824 Lag3: 0.879 Lag4: 0.913	Lag1: 0.577 Lag2: 0.803 Lag3: 0.815 Lag4: 0.852	Lag1: 0.537 Lag2: 0.813 Lag3: 0.896 Lag4: 0.899
White test for homoscedasticity (H0: homoscedastic residuals); p-values	0.552	0.242	0.380	0.511
Cumulative Sum test for Parameters stability (H0); p-values	0.488	0.418	0.420	0.412

Note: Significance levels -

***, 1%

**, 5%

*, 10%, following PSS (2001) Below the label of each country, there appears between parentheses the preferred specification for the ARDL model run considering the Bayesian information criterion.

Table 3 shows how our series are heterogeneous considering their characteristics of stationarity. Considering an overall insight, we claim that first-differences of our series are stationary at a significance level below 1% and if considering the interception parameter in the test equation.

Considering the results from Table 4, several insights emerge. First, the estimated adjustment parameters (λ) have been found as statistically significant at a 1% significance level which suggests the existence of long adjustments. For instance, for Brazil, the adjustment is approximately 20 time units (1/0.054), in our case, 20 months. Similar interpretations can be extended to the models estimated for Colombia, Mexico and Peru. This follows authors such as Islam et al. (2012) and Rezgar (2015), who also found additional long adjustments in their works. Regarding the stability issue of the estimated residuals for the cointegration regressions, all of the usual tests suggest the presence of stable residuals (full details available upon request).

For the long-run estimates, some challenges arise. First, we found the expected positive and significant coefficients estimated for inflation rates in Colombia (+0.882) and Mexico (+0.886), suggesting that in the long run, rises in the inflation rates of these economies tended to motivate rises in the observed national interest rates (for each 1% rise, there is an expectation of a 0.9% rise in the interest rate of these two countries). However, non-statistically significant coefficients were found for Brazil (-2.433) and for Mexico (-7.076), at a 5% level. Therefore, we can claim that in the long run, there has been independence of Brazilian and Peruvian interest rates from national inflation levels. The output gap has also no effect on the long-term evolution of interest rates in these economies according to Table 4.

Short-run estimates associate the reaction observed in the monthly change rate of the interest rates due to the respective change in each independent variable. Table 4 reveals some interesting issues. Short-term stimuli from monthly changes in the inflation rate conducted to changes in the interest rates observed for each economy. These stimuli have been found statistically significant (at 1% level), ranging from 0.114 (Brazil) to 0.509 (Colombia). Once again, the output gap has been found as not having estimated coefficients with statistically significant values.

Finally, we also tested critical values and approximate p-values based on response surface regressions from Kripfganz and Schneider (2018)/KS for the Pesaran et al (2001) bounds testing procedure. For all of the estimated error-correction models, we obtained values that allowed us to reject the respective null hypothesis (of no cointegration in the defined relations between the interest rates, as dependent variable, and inflation rates or output gaps, as independent variables). The traditional Breusch-Godfrey serial correlation test, the White test for heteroscedasticity, and the CUSUM test (its cumulative version) have been employed to verify the stability of long-run coefficients together with short-run dynamics. These values are exhibited in the last rows of Table 4, assessing the necessary properties for inference upon the estimation.

However, following Mourao and Stawska (2020) [51], there is an established tradition in time series analysis (Hamilton, 1994) suggesting that Markov switching models should be used to analyze periods of significant differences. These models have been found to be useful for identifying different states (also named ‘regimes’) in the observed time series.

Following the most common notation according to Markov switching models, we start our discussion by considering a time series y_t_ (observed from period t=1,…,T) modeled by K number of states. Let us assume two states (K=2), without loss of generalization. Therefore, yt can be differently modeled for State 1 and for State 2.

For State 1, yt is modeled as Eq 3

State1:yt=μ1+εt (Eq 3)

For State 2, yt is modeled as Eq 4

State2:yt=μ2+εt (Eq 4)

In Eqs 3 and 4, μ1 and μ2 are the intercept terms in state 1 and state 2, respectively. εt is assumed to follow standard assumptions: a white noise error with variance σ2. The two-states model differs in the intercept terms (μ1 and μ2).

In a more general terminology, we can model Eqs 2 and 3 as a Markov-switching regression model.

Markov-switching regression models will let the parameters vary over the unobserved states:

$$y_{t}=\mu_{st}+\varepsilon_{t}$$

where μ_st_ is the parameter of interest; μ_st_ = μ_1_ when the observed state is of type 1 (st = 1), and μ_st_ = μ_2_ when the state is of type 2 (st = 2).

For estimating st, the conventional procedure is based on transition probabilities, which for K=2 are estimated in a matrix as follows:

$$\left[\begin{array}{cc} p11 & p21\\ p12 & p22 \end{array}\right]$$ (Matrix 1)

According to Krolzig (1997) [57], p11 denotes the probability of the series yt staying in state 1 in the next period given that yt is in state 1 in the current period. p12 relates to the probability of moving to state 2 after a period characterized by being in state 1. Conversely, p21 exhibits the estimated probability of transitioning to state 1 after a period in state 2, and p22 denotes the probability of staying in state 2. More persistent processes are characterized by estimated probabilities close to 1.

Markov-switching dynamic regressions (MSDRs) are also common estimation methods (for testing the statistical significance of the different states, of the state-dependent means, or the error variance). Different specifications of Markov-switching dynamic regressions allow us to test the statistical significance of switching intercepts and coefficients, exogenous variables, and switching variances.

As also discussed by Krolzig (1997) [57], for measuring gradual adjustment after the process changes from one state to another, Markov-Switching autoregressive (AR) models may also be estimated and analyzed without losing the possibility of extending the analysis to switching intercepts, coefficients, exogenous variables, and switching variances. To discuss the preference for certain specifications, such as the number of states among other features of the models’ specification, criteria such as AIC, BIC or SBIC are followed.

Table 5 shows our estimations of MS regressions on the presence of Taylor rules related to the four observed southern American economies since 1995.

**Table 5:** MS regressions on Taylor rule.

	Brazil	Colombia	Mexico	Peru
**State 1**				
Interest rates (lag)	0.7323*** (0.024)	0.833*** (0.034)	0.892*** (0.011)	0.456*** (0.031)
Inflation	0.044*** (0.005)	0.155* (0.083)	0.304** (0.156)	6.776*** (0.401)
Output gap	-2e-5 (2e-3)	0.014** (0.005)	0.001 (0.001)	-0.651*** (0.079)
Constant	3.300*** (0.488)	-0.141 (0.226)	0.655*** (0.098)	2.961*** (0.498)
**State 2**				
Interest rates (lag)	0.961*** (0.024)	0.944*** (0.012)	-1.012*** (0.119)	1.575*** (0.056)
Inflation	0.161*** (0.017)	0.058*** (0.021)	30.271*** (2.497)	-4.509*** (0.280)
Output gap	0.002 (0.001)	9e-4 (7e-3)	-0.061 (0.041)	-0.634*** (0.132)
Constant	-0.325 (0.359)	0.104* (0.059)	17.387 (0.772)	-4.511*** (0.911)
**State 1**				
Expected Duration	1.192 (0.166)	5.794 (2.441)	38.201 (14.782)	1.677 (0.377)
Most-likely periods	1995m1–2000m1	2002m1–2002m12; 2009m1–2009m12; 2013m1–2015m12; 2017m1–2018m12	2000m1–2018m12	1995m1–1999m12
**State 2**				
Expected Duration	2.596 (0.947)	25.459 (14.001)	1.690 (0.430)	24.454 (7.684)
Most-likely periods	2000m1–2020m1	2000m1–2001m12; 2003m1–2009m1; 2010m1–2013m12; 2015m1–2017m12; 2018m1–2020m1	1995m1–1999m12	1999m1–2020m1

Note: Significance level -

***, 1%

**, 5%

*, 10%.

Considering estimates from Table 3, we can add the following insights. Most periods characterized by higher interest rates observed in the studied four Latin American economies have been estimated in the period before 2000 and coincide with the identified “State 1” for Brazil, Colombia, and Peru and with the identified “State 2” for Mexico.

Across the various columns of Table 4, we can check that interest rates were particularly reactive to inflation moves, reinforcing the idea of the presence of Taylor policies in the observed economies in the analyzed period. The coefficients estimated for inflation were particularly significant in State 2 for most of these economies, suggesting the latency of Taylor rules in the implementation of national monetary policies in these countries (especially since 2000).

Overall, the estimated duration for State 2 (in Brazil, Colombia, and Peru) or for State 1 in Mexico suggests the existence of a long period of stabilized interest rates in these four economies since 2000 which converge to the found low speeds of adjustment in Table 3.

Now, we will deepen our analysis by involving the political cycle. It is expected that in a political cycle focused on the clear application of the Taylor rule, there will be the coincidence of a reduction in interest rates following the reduction in inflation rates as well as in the stimuli from the output gap.

Thus, should certain incumbencies receive acknowledgement for the stabilization’s achievements or do stabilization trends follow independently of governments (leaving some responsibility to exogenous forces and to national dynamics independent of the political cycle)? To address this question, we will analyze the presence of structural breaks in each series observed for the four economies since 1995. The existence of structural breaks associated with a given government favors the hypothesis of the responsibility of that government as a modifier of the structure of the series in question.

As Mourao (2018) [58] states, “The history of the analysis of structural breaks in time series is well documented in works like Aue and Horvath (2013) or Lu and Ito (2008) [59,60]. From the first generations, focused on testing the statistical significance of structural breaks identified for precise moments (like the Chow test), we now have tests for unknown dates. Within these modern tests, we find the tests for multiple time breaks, like Clemente et al. (1998) [61], whose critical values were previously suggested by Perron and Vogelsang (1992) [62].”

The test of Clemente et al. (1998) [61] allowed us to analyze the nature of breaks, differentiating between sudden breaks in the series (‘additive outliers’) and smooth changes (‘innovational outliers’). Mourao and Martinho (2016) [63] reported that tests such as that of Clemente et al. (1998) [61] have additional convenience properties because they do not have as many restrictions on the stationarity of series as tests such as Bai and Perron (2003) [37], which imposed, for instance, that the series must be I(0), i.e., stationary at certain levels.

Using the forms of Baum (2005) [64], we make b_t_ refer to our series observed for each of the 4 observed Latin American countries (Brazil, Peru, Mexico, and Colombia). Therefore, we will observe for each of these countries – and for the period 1995:m1 to 2018:m12 – the following series:

- Nominal interest rates.
- Consumer price index.
- GDP output gap (measured considering the Hodrick-Prescott filter).

To test the presence of multiple additive outliers, we estimate the following system of Eq 5:

$$\begin{array}{l} b_{t}=\alpha +\delta_{1}DU_{1t}+\delta_{2}DU_{2t}+e_{t}\\ e_{t}=\sum_{i=1}^{k}w_{1i}DT_{b1,t-i}+\sum_{i=1}^{k}w_{2i}DT_{b2,t-i}+\rho e_{t-i}+\sum_{i=1}^{k}\theta_{i}\Delta e_{t-i}+z_{t} \end{array}$$ (Eq 5)

DU_1t_ = 1 for year t after the first break time and zero otherwise. Equivalently, DU_2t_ is equal to 1 for time observation t after the second break time and zero otherwise (Mourao, 2018). T_b1_ and T_b2_ represent the break points to be analyzed by grid search (i.e., by identifying the minimal t-ratio for the hypothesis *ρ* = 1). Following Baum (2005) and Mourao and Stawska (2020) [51,64], we use DT_bm,t_ = 1 for t = T_bm + 1_ and 0 for m = 1, 2.

To test *ρ* = 1 with the presence of innovational outliers, we analyze the model provided by Eq 6 (Baum, 2005) [64]:

$$\begin{array}{l} b_{t}=\alpha +\delta_{1}DU_{1t}+\delta_{2}DU_{2t}+w_{1}DT_{b1,t}+w_{2}DT_{b2,t}+\alpha b_{t-i}+\sum_{i=1}^{k}\theta_{i}\Delta b_{t-i}+z_{t} \end{array}$$ (Eq 6)

Now, we will discuss and detail the profile of each series, focusing on the identified structural breaks (Table 3). We also estimated Markov-switching for each individual series. We estimated the following models (MS dynamic regression; MSDR switching coefficients; MSDR switching variances; and MS autoregressive models). We will skip the full details of these specifications (which are available upon request). For comparison purposes, the preferred model for each series of the set of 4 Latin American countries we are studying and considering with respect to SBIC are the following.

For Brazil: nominal interest rates, MS dynamic regression (SBIC = -1.722); consumer price index, MS dynamic regression (SBIC= - 3.2355); output gap, MS dynamic regression (SBIC= -1.652).

For Colombia: nominal interest rates, MS dynamic regression (SBIC = -1.872); consumer price index, MS dynamic regression (SBIC= - 2.762); output gap, MS AR (SBIC= -4.221).

For Mexico: nominal interest rates, MSDR switching coefficients (SBIC = -3.261); consumer price index, MS dynamic regression (SBIC= - 4.299); output gap, MS dynamic regression (SBIC= -3.012).

For Peru: nominal interest rates, MSDR switching coefficients (SBIC = -2.081); consumer price index, MS dynamic regression (SBIC= - 2.001); output gap, MS AR (SBIC= -1.322).

Following this modeling, for each year, we obtained estimates of the filtered transition probabilities, which led us to identify the most likely state for each year for each series. Full details are available upon request (namely, duration of the states, their mean values at each series, and the respective transition probabilities).

The columns of Table 6 also identify the most likely state considering Markov-switching regressions for our series as well as the presence of a structural break of the identified series considering each identified government.

**Table 6:** Identification of significant changes in nominal interest rates, inflation and output gaps in Brazil, Colombia, México, and Peru (1995m1–2018m12).

	Nominal Interest Rates	Inflation	Output gap	Ruling Party	Period	President
***Brazil***		++		Partido da Social Democracia Brasileira (PSDB)	1995–2002	Fernando Henrique Cardoso
	--	--		Partido dos Trabalhadores (PT)	2003–2010	Luiz Inácio Lula da Silva
			++	Partido dos Trabalhadores (PT)	01/01/2011 - 31/08/2016	Dilma Rousseff
				Movimento Democrático Brasileiro (MDB - old PMDB)	31/08/2016 - 01/01/2019	Michel Temer
***Colombia***		--		Liberal	1994–1998	Ernesto Samper Pizano
	--			Conservador	1998–2002	Andrés Pastrana Arango
	--	--		Primero Colombia	2002–2010	Álvaro Uribe Vélez
			NS	Partido de la U	2010–2018	Juan Manuel Santos Calderón
***Peru***	-	NS		Mudança 90 e Perú 2000	05/04/1992 - 21/11/2000	Alberto Fujimori Fujimori
		-		Partido de la Acción Popular	22/11/2000 -28/08/2001	Valentín Paniagua Corazao
	--			Perú Posible	28/07/2001 -28/07/2006	Alejandro Toledo Manrique
			NS	Alianza Popular Revolucionaria Americana (PARA)	28/07/2006 - 28/07/2011	Alan García Pérez
				Partido Nacionalista Peruano	28/07/2011 - 28/07/2016	Ollanta Moisés Humala Tasso
				Peruanos Por el Kambio (PPK)	28/07/2016 - 23/03/2018	Pedro Pablo Kuczynski Godard
				Peruanos Por el Kambio (PPK)	23/03/2018 - actual	Martín Alberto Vizcarra Cornejo
***México***	--	+		Partido Revolucionário Institucional (PRI)	1994–2000	Ernesto Zedillo Ponce de León
	--		++	Partido Acción Nacional (PAN)	2000–2006	Vicente Foz Quesada
			++	Partido Acción Nacional (PAN)	2006–2012	Felipe Calderón Hinojosa
				Partido Revolucionário Institucional (PRI)	2012–2018	Henrique Peña Nieto

++/--: period of significant increases (/decreases) identified by structural breaks and by significant Markov states. +/-: period of significant increases (/decreases) only identified by structural breaks. NS: period of changes identified by structural breaks but without statistical significance. Blank cells indicate political periods without the identification of significant structural breaks or of Markov state changes.

Table 6 synthesizes the direction of our estimations (available upon request). As in the legend, we identified for each country the periods simultaneously characterized by significant structural breaks and significant Markov states. Considering breaks of positive/negative directions (+/-) and states of low/high (-/+) values in the series, we have four different combinations of these possibilities. If the definition of a Markov state was not found to be significant by our preferred model of Markov regressions, we only exhibit the identification from the test for the structural breaks, if the latter is significant; otherwise, we identify with NS those political periods in which our tests identified structural breaks in the suggested period without statistical significance. A blank cell indicates political periods without the identification of significant structural breaks or of Markov state changes.

As noted in Table 6, we see with greater clarity that the Taylor rule was associated with the cases of Brazil between 2003 and 2010 and Colombia between 2002 and 2010. Being focused on the reality of each country, Brazil had a significant reduction in interest rates between 2003 and 2010 accompanied by a reduction in the inflation rate. In this period, both movements were identified by the tests of structural breaks and in the Markov states models. In contrast, between 1995 and 2002, both procedures identified growth in inflation. There was also an increase in the output gap (signaling economic growth) by the two empirical tests for 2011 and 2016.

For Colombia, there was a reduction in inflation between 1994 and 1998 identified by both procedures as well as a reduction in interest rates between 1998 and 2002 and between 2002 and 2010.

Within the sample period analyzed for Peru, there was a reduction in nominal interest rates between 1992 and 2000 and in the inflation rate between 2000 and 2001, only observed by the structural breaks tests. The period between 2001 and 2006, on the other hand, was a time when both methodological procedures (structural breaks tests and Markov’s state changes) identified a reduction in Colombian interest rates.

On the other hand, Mexico had more changes simultaneously identified by both methodological procedures. In fact, the structural breaks and Markov states detected a reduction in nominal interest rates between 1994 and 2000 and between 2000 and 2006. The same procedures detected increases in the output gap between 2000 and 2006 and between 2006 and 2012. There was still a period of growth in inflation between 1994 and 2000 detected by the structural break tests.

In summary, our procedures robustly clarify the observation of Taylor’s rule at specific moments in the observed period. In this case, it was observed that Taylor’s rule was associated with the cases of Brazil between 2003 and 2010 and Colombia between 2002 and 2010. For Peru and Mexico, there was also, in general, a reduction in nominal interest rates, but the other variables usually considered in the Taylor rule (inflation and output gap) were not validated by all tests. We also noticed that within the sample period (1995m1–2018m12), there was a determined control of the nominal interest rate in the initial moments. Regarding the profile of the evolution of the inflation rate, a reduction in the trend was followed, although in certain cases – as in Brazil – there had been an initial and significant growth.

The output gap was the variable in Table 5 identified as having experienced the least significant changes in each political period. With the exception of Mexico under the majority of the PAN (2000 to 2012) and Brazil (2011 to 2016), most other periods and other countries did not register structural changes in this variable, which tends to be associated with economic growth (if the gap is a positive value) or associated with economic crisis (if the gap is negative).

## Discussion, conclusions and policy implications

This work revisited one of the most intense debates in macroeconomics in recent decades: the presence of the Taylor rule in the implementation of monetary policy in Latin America in the last forty years. During the 1960s and 1970s, various Latin American countries experienced a reality of accumulated external debt that generated intense crises after 1980, with visible consequences of high levels of inflation, emerging social tensions, the devaluation of the formal economy and the depreciation of national currencies on international markets.

The political generation that emerged in the 1990s coincided with the period after these crises. This is a period associated with the application of the Taylor rule (that is, with the indexation of the national interest rate to the evolution of pressure on prices, reflected in inflation and in the output gap). In that period, a debate emerged that has been prolonged to the present. Basically, this debate seeks to discuss the greater responsibility for fixing these episodes of inflationary crisis and public debt. A stream of authors has highlighted the role of international exogenous forces in helping to overcome economic crisis. Another trend has pointed out the importance of endogenous policies and certain governments for this result.

Through appropriate econometric procedures, we evaluated such claims. Through ARDL, Markov Switching models, and structural breaks, we observed the behavior of the series of nominal interest rates, inflation rates and the output gap for four economies: Brazil, Colombia, Mexico and Peru. These series were observed between 1990m1 and 2018m12. The results achieved are very stimulating. On one hand, they identify some governments in the region coinciding with structural changes in the series, reinforcing the role that adequate governance in the region can play in effectively conducting economic and monetary policy. On the other hand, the observation for most economies was that the presence of a longer state coincided with the observations after 2000 where the implementation of the interest policy was mainly associated with the evolution of the inflation rate.

From the achieved results, we recognize the presence of three economic and monetary policy implications as well as society’s benefits. First, the relationship between certain political cycles and monetary stabilization cycles shows that political direction matters for the success of monetary stabilization. Second, monetary stabilization processes tended to be more successful after the year 2000, coinciding with a period of long stabilization after initial periods of greater turmoil. Thus, it is shown that monetary stabilization rules such as the Taylor rule must be modeled as stable relationships in the long run, with very unstable moments in the periods when regulation begins. Finally, there is the recognition of a more significant relationship between interest rates and inflation rates, without a special influence of the output gap in Latin American economies. In the observations carried out, there tended to be a significant reduction in inflation rates, in the first place, and in subsequent political cycles, a significant reduction in interest rates. This type of transition suggests that, in the observed economies, there were initial mandates that were focused on controlling inflationary pressures and only the following political mandates managed to succeed in stabilizing interest rates. Regarding the societal benefits of this investigation, we identified two eminently. First, this work reinforced the need for political stabilization as a factor in monetary stabilization. Secondly, this work also demonstrated how governments tend to start stabilization processes from the control of inflation rates as a dimension that, also a priori, implies greater social impacts.

There are paths for future research derived from this investigation’s limitations. We can enunciate these limitations as related to the Latin-America representativeness, to the focus on presidential cycles, to the absence of detail on recent monetary crisis, and to the restriction to the conventional versions of the Taylor-rule or of the interest rates. So, on the one hand, we want to expand the range of observed countries and extend the observed time series as much as possible. Another emerging challenge is related to a greater detail of the political cycle, which in the observed countries is generally identified with the presidential cycle. Thus, we intend to detail the political cycle by finance ministers and by economy ministers from each of the region’s economies. We also intend to revise the paper considering the possibility of significant changes coming from the 2008 crisis. In the literature, we also can find forward-looking versions of the Taylor rule; therefore, another challenge emerges from the possibility of considering these more complex versions of the Taylor rule in our theoretical and empirical models. Finally, we intend to explore the different interest rates observed in each economy and explore the reaction by different price indices (not only the price indices realized by consumers but also producer price indices, additionally differentiating by sector of activity).

### Compliance with ethical standards

#### Ethical approval

This article does not contain any studies with human participants performed by any of the authors.
